# 4-Chloro-2-[(2,6-diisopropyl­phen­yl)imino­meth­yl]phenol

**DOI:** 10.1107/S1600536812020612

**Published:** 2012-05-16

**Authors:** P. Balamurugan, K. Kanmani Raja, D. Easwaramoorthy, G. Chakkaravarthi, G. Rajagopal

**Affiliations:** a Department of Chemistry, Government Arts College (Men), Nandanam, Chennai 600 035, India; b Department of Chemistry, Government Thirumagal Mills College, Gudiyattam 632 604, India; c Department of Chemistry, B.S. Abdur Rahman University, Vandalur, Chennai 600 049, India; d Department of Physics, CPCL Polytechnic College, Chennai 600 068, India; e Department of Chemistry, Government Arts College, Melur 625 106, India

## Abstract

The asymmetric unit of the title compound, C_19_H_22_ClNO, contains two independent mol­ecules in which the dihedral angles between the aromatic rings are 76.45 (9) and 74.69 (9)°. An intra­molecular O—H⋯N hydrogen bond occurs in each mol­ecule. The crystal structure features weak C—H⋯π inter­actions.

## Related literature

For the biological activity of Schiff base ligands, see: Santos *et al.* (2001 ▶). For related strucutures, see: Raja *et al.* (2008 ▶); Lin *et al.* (2005 ▶).
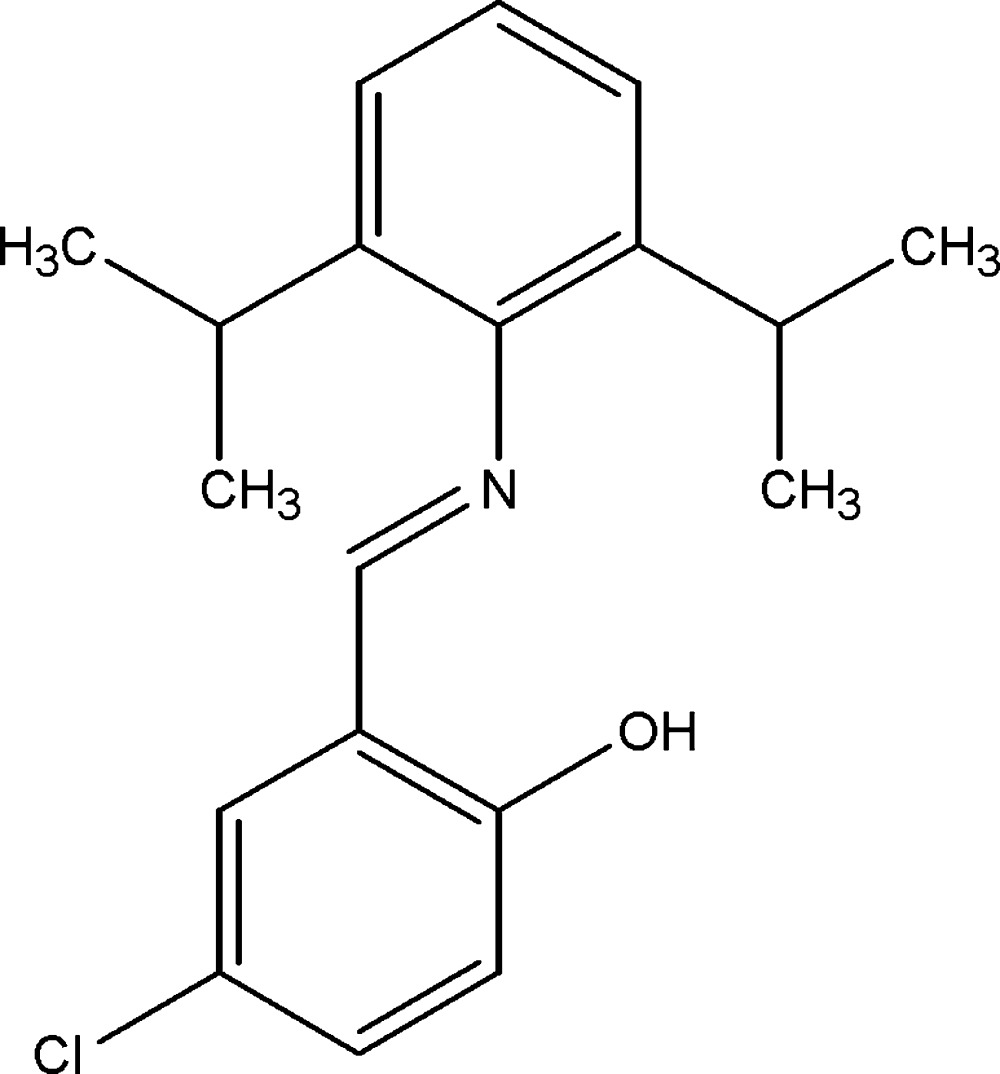


## Experimental

### 

#### Crystal data

C_19_H_22_ClNO
*M* *_r_* = 315.83
Triclinic, 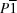

*a* = 11.276 (2) Å
*b* = 11.776 (2) Å
*c* = 14.189 (3) Å
α = 73.01 (3)°
β = 88.42 (2)°
γ = 85.57 (3)°
*V* = 1796.5 (6) Å^3^
*Z* = 4
Mo *K*α radiation
μ = 0.21 mm^−1^
*T* = 295 K
0.30 × 0.20 × 0.16 mm

#### Data collection

Bruker Kappa APEXII diffractometer
Absorption correction: multi-scan (*SADABS*; Sheldrick, 1996 ▶) *T*_min_ = 0.939, *T*_max_ = 0.967
40436 measured reflections
8907 independent reflections
5518 reflections with *I* > 2σ(*I*)
*R*_int_ = 0.027

#### Refinement

*R*[*F* ^2^ > 2σ(*F* ^2^)] = 0.049
*wR*(*F* ^2^) = 0.153
*S* = 1.02
8907 reflections
407 parameters
H-atom parameters constrained
Δρ_max_ = 0.30 e Å^−3^
Δρ_min_ = −0.36 e Å^−3^

### 

Data collection: *APEX2* (Bruker, 2004 ▶); cell refinement: *SAINT* (Bruker, 2004 ▶); data reduction: *SAINT*; program(s) used to solve structure: *SHELXS97* (Sheldrick, 2008 ▶); program(s) used to refine structure: *SHELXL97* (Sheldrick, 2008 ▶); molecular graphics: *PLATON* (Spek, 2009 ▶); software used to prepare material for publication: *SHELXL97*.

## Supplementary Material

Crystal structure: contains datablock(s) global, I. DOI: 10.1107/S1600536812020612/vm2174sup1.cif

Structure factors: contains datablock(s) I. DOI: 10.1107/S1600536812020612/vm2174Isup2.hkl

Supplementary material file. DOI: 10.1107/S1600536812020612/vm2174Isup3.cml

Additional supplementary materials:  crystallographic information; 3D view; checkCIF report

## Figures and Tables

**Table 1:** Hydrogen-bond geometry (Å, °) *Cg*1 and *Cg*3 are the centroids of the C1–C6 and C20–C25 rings, respectively.

*D*—H⋯*A*	*D*—H	H⋯*A*	*D*⋯*A*	*D*—H⋯*A*
O1—H1⋯N1	0.82	1.92	2.646 (2)	147
O2—H2⋯N2	0.82	1.90	2.630 (2)	147
C31—H31c⋯*Cg*1	0.96	2.90	3.743 (3)	147
C12—H12*A*⋯*Cg*3^i^	0.96	2.98	3.833 (3)	149

Symmetry code: (i) 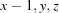.

**Fig. 1.**
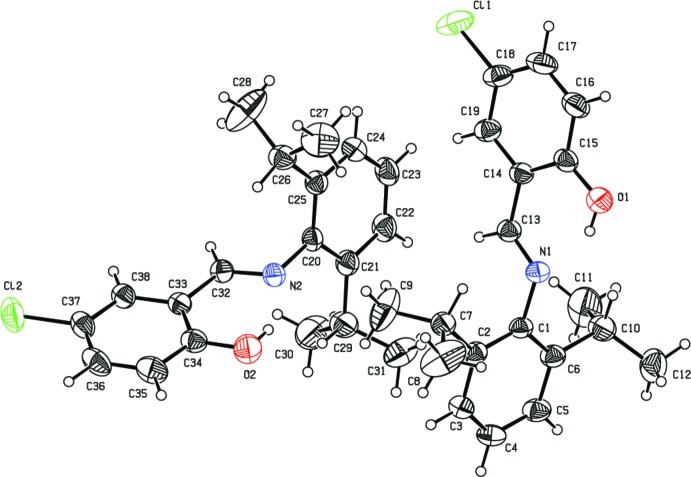
The molecular structure of (I), with atom labels and 30% probability displacement ellipsoids for non-H atoms.

**Fig. 2.**
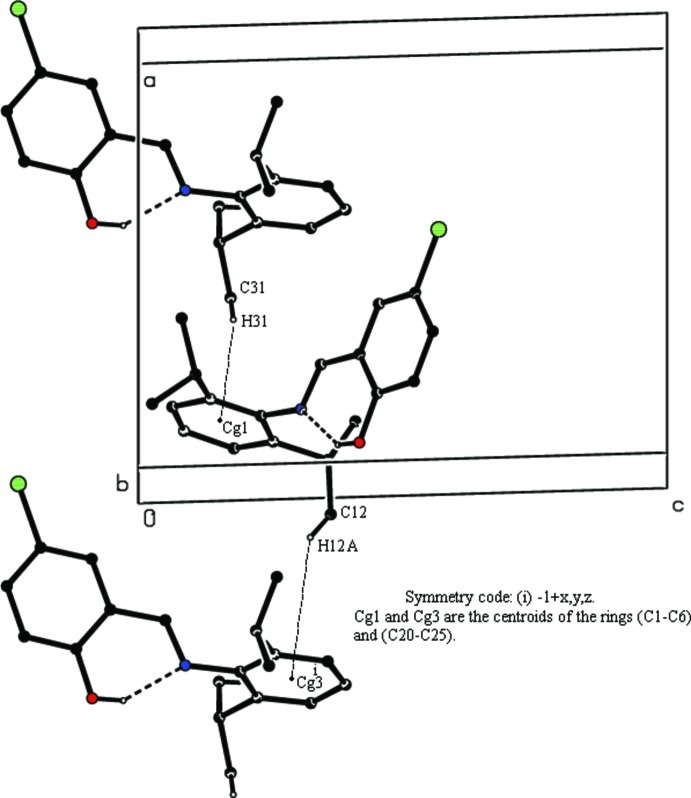
The packing of (I), viewed down b axis. Intramolecular hydrogen bond and C-H···π interactions are shown as dashed lines. H atoms not involved in hydrogen bonding have been omitted. [Symmetry code (i) -1 + x, y, z. Cg1 and Cg3 are the centroids of the rings (C1-C6) and (C20-C25)].

## supplementary crystallographic information

### Comment

Schiff base derivatives are found to exhibit important pharmacological properties, such as antibacterial, antitumor and antitoxic activities (Santos *et al.*, 2001).

The asymmetric unit of the title compound (I) (Fig. 1) contains two molecules. The geometric parameters in (I) are comparable with the similar reported structures (Raja *et al.*, 2008; Lin *et al.*, 2005). The dihedral angles between the aromatic rings (C1-C6) and (C14-C19) & (C21-C26) and (C33-C38) are 76.45 (9) and 74.69 (9)°, respectively. In the asymmetric unit of (I), both molecules adopt an anti-periplanar conformation [C14-C13-N1-C1 = 176.31 (15)° and C33-C32-N2-C20 = -176.22 (15)°] about the C=N bond.

The molecular structure is stabilized by weak intramolecular O-H···N hydrogen bonds and C-H···π interactions (Table 1). The crystal structure is stabilized by a weak intermolecular C-H···π interaction (Table 1).

### Experimental

An ethanolic solution (10 ml) of 2,6-diisopropylaniline [2 mmol] was magnetically stirred in a round bottom flask followed by drop wise addition of ethanolic solution (10 ml) of 5-chlorosalicylaldehyde [2 mmol]. The reaction mixture was then refluxed for 3 h and upon cooling to 273 K, a yellow solid precipitated from the reaction mixture. The solid which separated out was filtered, washed with ice cold ethanol and dried over anhydrous CaCl_2_. Single crystals of good diffraction quality were obtained by recrystallization of the compound from an ethanol solution by the slow evaporation method.Yield: 70 %.

### Refinement

H atoms were positioned geometrically with C—H = 0.93–0.97 Å and O–H = 0.82 Å and allowed to ride on their parent atoms, with Uiso(H) = 1.5 U_eq_(O) or 1.2U_eq_(C) or 1.5U_eq_(C_methyl_).

### Crystal data

**Table:**

C_19_H_22_ClNO	*Z* = 4
*M**_r_* = 315.83	*F*(000) = 672
Triclinic, *P*1	*D*_x_ = 1.168 Mg m^−^^3^
Hall symbol: -P 1	Mo *K*α radiation, λ = 0.71073 Å
*a* = 11.276 (2) Å	Cell parameters from 8910 reflections
*b* = 11.776 (2) Å	θ = 1.5–28.4°
*c* = 14.189 (3) Å	µ = 0.21 mm^−^^1^
α = 73.01 (3)°	*T* = 295 K
β = 88.42 (2)°	Prism, light yellow
γ = 85.57 (3)°	0.30 × 0.20 × 0.16 mm
*V* = 1796.5 (6) Å^3^	

### Data collection

**Table:**

Bruker Kappa APEXII diffractometer	8907 independent reflections
Radiation source: fine-focus sealed tube	5518 reflections with *I* > 2σ(*I*)
Graphite monochromator	*R*_int_ = 0.027
ω and φ scans	θ_max_ = 28.4°, θ_min_ = 2.0°
Absorption correction: multi-scan (*SADABS*; Sheldrick, 1996)	*h* = −15→15
*T*_min_ = 0.939, *T*_max_ = 0.967	*k* = −15→15
40436 measured reflections	*l* = −18→18

### Refinement

**Table:**

Refinement on *F*^2^	Primary atom site location: structure-invariant direct methods
Least-squares matrix: full	Secondary atom site location: difference Fourier map
*R*[*F*^2^ > 2σ(*F*^2^)] = 0.049	Hydrogen site location: inferred from neighbouring sites
*wR*(*F*^2^) = 0.153	H-atom parameters constrained
*S* = 1.02	*w* = 1/[σ^2^(*F*_o_^2^) + (0.0607*P*)^2^ + 0.4833*P*] where *P* = (*F*_o_^2^ + 2*F*_c_^2^)/3
8907 reflections	(Δ/σ)_max_ < 0.001
407 parameters	Δρ_max_ = 0.30 e Å^−^^3^
0 restraints	Δρ_min_ = −0.36 e Å^−^^3^

### Fractional atomic coordinates and isotropic or equivalent isotropic displacement parameters (Å^2^)

**Table:**

	*x*	*y*	*z*	*U*_iso_*/*U*_eq_	
C1	0.13739 (14)	0.66107 (15)	0.23173 (12)	0.0498 (4)	
C2	0.17448 (14)	0.71891 (15)	0.13594 (13)	0.0509 (4)	
C3	0.16389 (16)	0.66098 (16)	0.06416 (14)	0.0584 (4)	
H3	0.1890	0.6969	0.0001	0.070*	
C4	0.11715 (18)	0.55202 (17)	0.08624 (15)	0.0652 (5)	
H4	0.1117	0.5143	0.0375	0.078*	
C5	0.07837 (18)	0.49844 (17)	0.18045 (15)	0.0658 (5)	
H5	0.0457	0.4251	0.1944	0.079*	
C6	0.08694 (16)	0.55152 (16)	0.25501 (13)	0.0572 (4)	
C7	0.22149 (17)	0.84165 (16)	0.10807 (14)	0.0602 (5)	
H7	0.2122	0.8713	0.1658	0.072*	
C8	0.1505 (3)	0.9267 (2)	0.0261 (3)	0.1324 (13)	
H8A	0.1773	1.0052	0.0141	0.199*	
H8B	0.0679	0.9276	0.0444	0.199*	
H8C	0.1608	0.9020	−0.0325	0.199*	
C9	0.3505 (2)	0.8389 (3)	0.0819 (3)	0.1291 (12)	
H9A	0.3952	0.7883	0.1370	0.194*	
H9B	0.3767	0.9180	0.0657	0.194*	
H9C	0.3627	0.8085	0.0263	0.194*	
C10	0.0474 (2)	0.48956 (19)	0.35955 (15)	0.0753 (6)	
H10	0.0405	0.5493	0.3955	0.090*	
C11	0.1404 (3)	0.3948 (3)	0.4105 (2)	0.1328 (12)	
H11A	0.1499	0.3350	0.3766	0.199*	
H11B	0.1161	0.3591	0.4773	0.199*	
H11C	0.2146	0.4297	0.4101	0.199*	
C12	−0.0733 (2)	0.4395 (2)	0.3639 (2)	0.0987 (8)	
H12A	−0.1291	0.4996	0.3252	0.148*	
H12B	−0.1005	0.4148	0.4310	0.148*	
H12C	−0.0668	0.3723	0.3382	0.148*	
C13	0.24570 (16)	0.71952 (15)	0.34528 (13)	0.0543 (4)	
H13	0.3115	0.6794	0.3255	0.065*	
C14	0.26162 (16)	0.78161 (15)	0.41832 (12)	0.0517 (4)	
C15	0.16653 (17)	0.84406 (16)	0.45179 (13)	0.0577 (4)	
C16	0.1866 (2)	0.9024 (2)	0.52119 (15)	0.0752 (6)	
H16	0.1238	0.9438	0.5436	0.090*	
C17	0.2994 (2)	0.8991 (2)	0.55676 (15)	0.0798 (7)	
H17	0.3126	0.9380	0.6035	0.096*	
C18	0.3930 (2)	0.83857 (18)	0.52351 (14)	0.0691 (6)	
C19	0.37495 (18)	0.78031 (16)	0.45505 (14)	0.0613 (5)	
H19	0.4388	0.7397	0.4331	0.074*	
C38	0.89501 (17)	0.76937 (15)	−0.08837 (13)	0.0574 (4)	
H38	0.9565	0.7309	−0.0459	0.069*	
C20	0.63784 (14)	0.65812 (15)	0.19237 (12)	0.0500 (4)	
C21	0.58671 (16)	0.54870 (16)	0.22519 (14)	0.0590 (4)	
C22	0.57451 (18)	0.49846 (18)	0.32620 (15)	0.0683 (5)	
H22	0.5417	0.4253	0.3498	0.082*	
C23	0.60990 (18)	0.55449 (19)	0.39185 (15)	0.0693 (5)	
H23	0.6033	0.5181	0.4592	0.083*	
C24	0.65504 (17)	0.66414 (18)	0.35815 (14)	0.0637 (5)	
H24	0.6762	0.7026	0.4032	0.076*	
C25	0.66973 (15)	0.71898 (16)	0.25773 (13)	0.0541 (4)	
C26	0.71529 (19)	0.84265 (17)	0.22278 (15)	0.0676 (5)	
H26	0.7221	0.8639	0.1508	0.081*	
C27	0.6271 (3)	0.9335 (2)	0.2472 (2)	0.1123 (9)	
H27A	0.6172	0.9144	0.3174	0.169*	
H27B	0.5520	0.9325	0.2173	0.169*	
H27C	0.6562	1.0112	0.2224	0.169*	
C28	0.8359 (3)	0.8457 (3)	0.2619 (3)	0.1506 (16)	
H28A	0.8322	0.8263	0.3324	0.226*	
H28B	0.8633	0.9239	0.2352	0.226*	
H28C	0.8899	0.7887	0.2432	0.226*	
C29	0.5491 (2)	0.4852 (2)	0.15336 (17)	0.0784 (6)	
H29	0.5484	0.5422	0.0873	0.094*	
C30	0.6386 (3)	0.3834 (3)	0.1526 (3)	0.1459 (15)	
H30A	0.7165	0.4123	0.1399	0.219*	
H30B	0.6184	0.3493	0.1020	0.219*	
H30C	0.6378	0.3239	0.2154	0.219*	
C31	0.4257 (2)	0.4417 (2)	0.1744 (2)	0.0997 (8)	
H31A	0.4262	0.3787	0.2353	0.150*	
H31B	0.4019	0.4125	0.1219	0.150*	
H31C	0.3708	0.5061	0.1795	0.150*	
C32	0.75544 (15)	0.71162 (14)	0.05083 (12)	0.0505 (4)	
H32	0.8191	0.6740	0.0912	0.061*	
C33	0.77830 (15)	0.76946 (14)	−0.05304 (12)	0.0496 (4)	
C34	0.68686 (18)	0.82811 (16)	−0.11783 (13)	0.0607 (5)	
C35	0.7139 (2)	0.8834 (2)	−0.21581 (16)	0.0809 (6)	
H35	0.6533	0.9218	−0.2594	0.097*	
C36	0.8297 (2)	0.88172 (19)	−0.24875 (15)	0.0791 (6)	
H36	0.8472	0.9190	−0.3145	0.095*	
C37	0.91965 (19)	0.82525 (17)	−0.18488 (15)	0.0647 (5)	
Cl1	0.53583 (7)	0.83560 (6)	0.56803 (5)	0.1042 (3)	
Cl2	1.06620 (6)	0.82311 (6)	−0.22699 (5)	0.0990 (2)	
N1	0.14538 (13)	0.71810 (13)	0.30767 (11)	0.0546 (3)	
N2	0.65162 (12)	0.71064 (12)	0.08848 (10)	0.0528 (3)	
O1	0.05522 (12)	0.85034 (14)	0.41805 (11)	0.0753 (4)	
H1	0.0543	0.8131	0.3775	0.113*	
O2	0.57289 (13)	0.83301 (16)	−0.08738 (11)	0.0832 (4)	
H2	0.5687	0.7966	−0.0287	0.125*	

### Atomic displacement parameters (Å^2^)

**Table:**

	*U*^11^	*U*^22^	*U*^33^	*U*^12^	*U*^13^	*U*^23^
C1	0.0504 (9)	0.0495 (9)	0.0544 (10)	−0.0034 (7)	−0.0043 (7)	−0.0226 (8)
C2	0.0494 (9)	0.0491 (9)	0.0567 (10)	−0.0026 (7)	−0.0023 (7)	−0.0196 (8)
C3	0.0671 (11)	0.0584 (11)	0.0526 (10)	−0.0031 (8)	−0.0008 (8)	−0.0210 (9)
C4	0.0814 (13)	0.0597 (11)	0.0642 (12)	−0.0053 (9)	−0.0102 (10)	−0.0325 (10)
C5	0.0825 (13)	0.0528 (10)	0.0684 (13)	−0.0161 (9)	−0.0052 (10)	−0.0241 (10)
C6	0.0649 (10)	0.0529 (10)	0.0575 (11)	−0.0108 (8)	−0.0024 (8)	−0.0199 (8)
C7	0.0686 (11)	0.0555 (10)	0.0603 (11)	−0.0140 (8)	0.0046 (9)	−0.0209 (9)
C8	0.147 (3)	0.0542 (14)	0.179 (3)	−0.0117 (15)	−0.063 (2)	−0.0002 (17)
C9	0.0758 (17)	0.096 (2)	0.216 (4)	−0.0333 (14)	0.026 (2)	−0.042 (2)
C10	0.0984 (16)	0.0677 (13)	0.0634 (12)	−0.0279 (11)	0.0082 (11)	−0.0199 (10)
C11	0.120 (2)	0.154 (3)	0.085 (2)	−0.005 (2)	−0.0113 (17)	0.0267 (19)
C12	0.0974 (18)	0.0964 (18)	0.1012 (19)	−0.0333 (14)	0.0262 (15)	−0.0225 (15)
C13	0.0599 (10)	0.0517 (10)	0.0543 (10)	−0.0074 (8)	−0.0005 (8)	−0.0192 (8)
C14	0.0655 (10)	0.0471 (9)	0.0436 (9)	−0.0151 (8)	−0.0012 (7)	−0.0117 (7)
C15	0.0745 (12)	0.0549 (10)	0.0449 (9)	−0.0179 (9)	0.0066 (8)	−0.0135 (8)
C16	0.0992 (16)	0.0766 (14)	0.0590 (12)	−0.0182 (12)	0.0127 (11)	−0.0321 (11)
C17	0.123 (2)	0.0747 (14)	0.0514 (12)	−0.0334 (14)	−0.0014 (12)	−0.0264 (11)
C18	0.0927 (15)	0.0597 (11)	0.0539 (11)	−0.0296 (11)	−0.0161 (10)	−0.0074 (9)
C19	0.0732 (12)	0.0546 (10)	0.0566 (11)	−0.0150 (9)	−0.0072 (9)	−0.0137 (9)
C38	0.0677 (11)	0.0507 (10)	0.0536 (10)	−0.0117 (8)	0.0064 (8)	−0.0135 (8)
C20	0.0473 (8)	0.0520 (9)	0.0467 (9)	−0.0048 (7)	0.0039 (7)	−0.0083 (7)
C21	0.0625 (11)	0.0558 (10)	0.0569 (11)	−0.0118 (8)	0.0061 (8)	−0.0122 (8)
C22	0.0752 (13)	0.0607 (12)	0.0636 (12)	−0.0174 (9)	0.0121 (10)	−0.0075 (10)
C23	0.0752 (13)	0.0746 (13)	0.0493 (11)	−0.0099 (10)	0.0096 (9)	−0.0039 (10)
C24	0.0684 (11)	0.0714 (12)	0.0529 (11)	−0.0073 (9)	0.0014 (8)	−0.0202 (9)
C25	0.0543 (9)	0.0543 (10)	0.0519 (10)	−0.0061 (7)	0.0014 (7)	−0.0125 (8)
C26	0.0854 (14)	0.0578 (11)	0.0624 (12)	−0.0177 (10)	0.0006 (10)	−0.0189 (9)
C27	0.140 (3)	0.0686 (16)	0.131 (2)	−0.0010 (16)	−0.008 (2)	−0.0328 (16)
C28	0.100 (2)	0.100 (2)	0.230 (4)	−0.0460 (17)	−0.046 (2)	0.000 (2)
C29	0.1006 (16)	0.0690 (13)	0.0709 (13)	−0.0313 (12)	0.0087 (12)	−0.0230 (11)
C30	0.098 (2)	0.178 (3)	0.218 (4)	−0.007 (2)	0.016 (2)	−0.148 (3)
C31	0.0865 (17)	0.0907 (17)	0.130 (2)	−0.0159 (13)	−0.0193 (15)	−0.0411 (16)
C32	0.0564 (9)	0.0456 (9)	0.0465 (9)	−0.0060 (7)	−0.0013 (7)	−0.0081 (7)
C33	0.0615 (10)	0.0412 (8)	0.0466 (9)	−0.0098 (7)	0.0019 (7)	−0.0122 (7)
C34	0.0724 (12)	0.0570 (11)	0.0516 (10)	−0.0075 (9)	−0.0044 (9)	−0.0130 (8)
C35	0.1028 (17)	0.0781 (15)	0.0524 (12)	0.0004 (12)	−0.0126 (11)	−0.0053 (10)
C36	0.122 (2)	0.0659 (13)	0.0458 (11)	−0.0168 (13)	0.0152 (12)	−0.0093 (10)
C37	0.0876 (13)	0.0505 (10)	0.0587 (11)	−0.0206 (9)	0.0217 (10)	−0.0183 (9)
Cl1	0.1159 (5)	0.1021 (5)	0.0971 (5)	−0.0390 (4)	−0.0434 (4)	−0.0211 (4)
Cl2	0.1058 (5)	0.1024 (5)	0.0922 (4)	−0.0357 (4)	0.0498 (4)	−0.0310 (4)
N1	0.0585 (8)	0.0561 (8)	0.0556 (9)	−0.0110 (6)	−0.0003 (6)	−0.0244 (7)
N2	0.0563 (8)	0.0521 (8)	0.0480 (8)	−0.0085 (6)	0.0024 (6)	−0.0107 (6)
O1	0.0675 (8)	0.0914 (11)	0.0773 (10)	−0.0064 (7)	0.0050 (7)	−0.0413 (8)
O2	0.0667 (9)	0.1046 (12)	0.0678 (9)	0.0040 (8)	−0.0107 (7)	−0.0104 (9)

### Geometric parameters (Å, º)

**Table:**

C1—C6	1.397 (2)	C38—C33	1.395 (2)
C1—C2	1.399 (2)	C38—H38	0.9300
C1—N1	1.435 (2)	C20—C25	1.396 (2)
C2—C3	1.394 (2)	C20—C21	1.400 (2)
C2—C7	1.516 (2)	C20—N2	1.431 (2)
C3—C4	1.372 (3)	C21—C22	1.389 (3)
C3—H3	0.9300	C21—C29	1.515 (3)
C4—C5	1.376 (3)	C22—C23	1.372 (3)
C4—H4	0.9300	C22—H22	0.9300
C5—C6	1.386 (3)	C23—C24	1.372 (3)
C5—H5	0.9300	C23—H23	0.9300
C6—C10	1.521 (3)	C24—C25	1.393 (3)
C7—C9	1.492 (3)	C24—H24	0.9300
C7—C8	1.498 (3)	C25—C26	1.520 (3)
C7—H7	0.9800	C26—C28	1.490 (3)
C8—H8A	0.9600	C26—C27	1.512 (3)
C8—H8B	0.9600	C26—H26	0.9800
C8—H8C	0.9600	C27—H27A	0.9600
C9—H9A	0.9600	C27—H27B	0.9600
C9—H9B	0.9600	C27—H27C	0.9600
C9—H9C	0.9600	C28—H28A	0.9600
C10—C11	1.502 (4)	C28—H28B	0.9600
C10—C12	1.517 (3)	C28—H28C	0.9600
C10—H10	0.9800	C29—C30	1.508 (4)
C11—H11A	0.9600	C29—C31	1.511 (3)
C11—H11B	0.9600	C29—H29	0.9800
C11—H11C	0.9600	C30—H30A	0.9600
C12—H12A	0.9600	C30—H30B	0.9600
C12—H12B	0.9600	C30—H30C	0.9600
C12—H12C	0.9600	C31—H31A	0.9600
C13—N1	1.267 (2)	C31—H31B	0.9600
C13—C14	1.454 (2)	C31—H31C	0.9600
C13—H13	0.9300	C32—N2	1.273 (2)
C14—C19	1.391 (2)	C32—C33	1.457 (2)
C14—C15	1.404 (3)	C32—H32	0.9300
C15—O1	1.345 (2)	C33—C34	1.399 (3)
C15—C16	1.388 (3)	C34—O2	1.347 (2)
C16—C17	1.375 (3)	C34—C35	1.388 (3)
C16—H16	0.9300	C35—C36	1.376 (3)
C17—C18	1.377 (3)	C35—H35	0.9300
C17—H17	0.9300	C36—C37	1.373 (3)
C18—C19	1.370 (3)	C36—H36	0.9300
C18—Cl1	1.740 (2)	C37—Cl2	1.743 (2)
C19—H19	0.9300	O1—H1	0.8200
C38—C37	1.366 (3)	O2—H2	0.8200
			
C6—C1—C2	121.94 (15)	C33—C38—H38	119.7
C6—C1—N1	118.55 (15)	C25—C20—C21	121.75 (16)
C2—C1—N1	119.43 (14)	C25—C20—N2	119.82 (14)
C3—C2—C1	117.57 (15)	C21—C20—N2	118.35 (16)
C3—C2—C7	119.87 (16)	C22—C21—C20	117.72 (17)
C1—C2—C7	122.54 (15)	C22—C21—C29	120.96 (17)
C4—C3—C2	121.24 (17)	C20—C21—C29	121.30 (17)
C4—C3—H3	119.4	C23—C22—C21	121.37 (18)
C2—C3—H3	119.4	C23—C22—H22	119.3
C3—C4—C5	120.05 (17)	C21—C22—H22	119.3
C3—C4—H4	120.0	C22—C23—C24	120.05 (18)
C5—C4—H4	120.0	C22—C23—H23	120.0
C4—C5—C6	121.33 (17)	C24—C23—H23	120.0
C4—C5—H5	119.3	C23—C24—C25	121.25 (18)
C6—C5—H5	119.3	C23—C24—H24	119.4
C5—C6—C1	117.82 (17)	C25—C24—H24	119.4
C5—C6—C10	120.80 (16)	C24—C25—C20	117.73 (16)
C1—C6—C10	121.33 (16)	C24—C25—C26	119.86 (17)
C9—C7—C8	110.6 (2)	C20—C25—C26	122.38 (16)
C9—C7—C2	112.29 (18)	C28—C26—C27	111.8 (2)
C8—C7—C2	111.16 (17)	C28—C26—C25	112.04 (19)
C9—C7—H7	107.5	C27—C26—C25	110.81 (19)
C8—C7—H7	107.5	C28—C26—H26	107.3
C2—C7—H7	107.5	C27—C26—H26	107.3
C7—C8—H8A	109.5	C25—C26—H26	107.3
C7—C8—H8B	109.5	C26—C27—H27A	109.5
H8A—C8—H8B	109.5	C26—C27—H27B	109.5
C7—C8—H8C	109.5	H27A—C27—H27B	109.5
H8A—C8—H8C	109.5	C26—C27—H27C	109.5
H8B—C8—H8C	109.5	H27A—C27—H27C	109.5
C7—C9—H9A	109.5	H27B—C27—H27C	109.5
C7—C9—H9B	109.5	C26—C28—H28A	109.5
H9A—C9—H9B	109.5	C26—C28—H28B	109.5
C7—C9—H9C	109.5	H28A—C28—H28B	109.5
H9A—C9—H9C	109.5	C26—C28—H28C	109.5
H9B—C9—H9C	109.5	H28A—C28—H28C	109.5
C11—C10—C12	111.2 (2)	H28B—C28—H28C	109.5
C11—C10—C6	110.4 (2)	C30—C29—C31	110.5 (2)
C12—C10—C6	112.97 (19)	C30—C29—C21	110.2 (2)
C11—C10—H10	107.3	C31—C29—C21	112.84 (19)
C12—C10—H10	107.3	C30—C29—H29	107.7
C6—C10—H10	107.3	C31—C29—H29	107.7
C10—C11—H11A	109.5	C21—C29—H29	107.7
C10—C11—H11B	109.5	C29—C30—H30A	109.5
H11A—C11—H11B	109.5	C29—C30—H30B	109.5
C10—C11—H11C	109.5	H30A—C30—H30B	109.5
H11A—C11—H11C	109.5	C29—C30—H30C	109.5
H11B—C11—H11C	109.5	H30A—C30—H30C	109.5
C10—C12—H12A	109.5	H30B—C30—H30C	109.5
C10—C12—H12B	109.5	C29—C31—H31A	109.5
H12A—C12—H12B	109.5	C29—C31—H31B	109.5
C10—C12—H12C	109.5	H31A—C31—H31B	109.5
H12A—C12—H12C	109.5	C29—C31—H31C	109.5
H12B—C12—H12C	109.5	H31A—C31—H31C	109.5
N1—C13—C14	122.37 (17)	H31B—C31—H31C	109.5
N1—C13—H13	118.8	N2—C32—C33	122.50 (16)
C14—C13—H13	118.8	N2—C32—H32	118.7
C19—C14—C15	119.09 (16)	C33—C32—H32	118.7
C19—C14—C13	118.86 (17)	C38—C33—C34	119.01 (16)
C15—C14—C13	122.04 (16)	C38—C33—C32	119.15 (16)
O1—C15—C16	118.21 (19)	C34—C33—C32	121.81 (16)
O1—C15—C14	122.01 (16)	O2—C34—C35	118.98 (19)
C16—C15—C14	119.79 (19)	O2—C34—C33	121.58 (16)
C17—C16—C15	119.9 (2)	C35—C34—C33	119.44 (19)
C17—C16—H16	120.0	C36—C35—C34	120.3 (2)
C15—C16—H16	120.0	C36—C35—H35	119.8
C16—C17—C18	120.36 (19)	C34—C35—H35	119.8
C16—C17—H17	119.8	C37—C36—C35	120.21 (19)
C18—C17—H17	119.8	C37—C36—H36	119.9
C19—C18—C17	120.6 (2)	C35—C36—H36	119.9
C19—C18—Cl1	119.25 (19)	C38—C37—C36	120.4 (2)
C17—C18—Cl1	120.18 (16)	C38—C37—Cl2	119.51 (18)
C18—C19—C14	120.3 (2)	C36—C37—Cl2	120.04 (16)
C18—C19—H19	119.9	C13—N1—C1	119.38 (15)
C14—C19—H19	119.9	C32—N2—C20	118.93 (15)
C37—C38—C33	120.55 (19)	C15—O1—H1	109.5
C37—C38—H38	119.7	C34—O2—H2	109.5
			
C6—C1—C2—C3	−2.6 (2)	N2—C20—C21—C29	1.4 (3)
N1—C1—C2—C3	−179.26 (15)	C20—C21—C22—C23	0.8 (3)
C6—C1—C2—C7	175.68 (16)	C29—C21—C22—C23	179.1 (2)
N1—C1—C2—C7	−1.0 (2)	C21—C22—C23—C24	2.1 (3)
C1—C2—C3—C4	1.0 (3)	C22—C23—C24—C25	−2.2 (3)
C7—C2—C3—C4	−177.32 (17)	C23—C24—C25—C20	−0.5 (3)
C2—C3—C4—C5	0.8 (3)	C23—C24—C25—C26	177.53 (18)
C3—C4—C5—C6	−1.0 (3)	C21—C20—C25—C24	3.4 (3)
C4—C5—C6—C1	−0.6 (3)	N2—C20—C25—C24	−179.81 (16)
C4—C5—C6—C10	−178.0 (2)	C21—C20—C25—C26	−174.56 (17)
C2—C1—C6—C5	2.4 (3)	N2—C20—C25—C26	2.2 (3)
N1—C1—C6—C5	179.10 (16)	C24—C25—C26—C28	60.4 (3)
C2—C1—C6—C10	179.81 (18)	C20—C25—C26—C28	−121.6 (3)
N1—C1—C6—C10	−3.5 (3)	C24—C25—C26—C27	−65.2 (3)
C3—C2—C7—C9	−69.4 (3)	C20—C25—C26—C27	112.7 (2)
C1—C2—C7—C9	112.4 (2)	C22—C21—C29—C30	−75.1 (3)
C3—C2—C7—C8	55.2 (3)	C20—C21—C29—C30	103.2 (3)
C1—C2—C7—C8	−123.0 (2)	C22—C21—C29—C31	49.0 (3)
C5—C6—C10—C11	78.3 (3)	C20—C21—C29—C31	−132.8 (2)
C1—C6—C10—C11	−99.1 (3)	C37—C38—C33—C34	−0.3 (3)
C5—C6—C10—C12	−46.9 (3)	C37—C38—C33—C32	−178.46 (16)
C1—C6—C10—C12	135.7 (2)	N2—C32—C33—C38	178.38 (16)
N1—C13—C14—C19	−179.12 (17)	N2—C32—C33—C34	0.3 (3)
N1—C13—C14—C15	0.0 (3)	C38—C33—C34—O2	−178.73 (17)
C19—C14—C15—O1	178.93 (16)	C32—C33—C34—O2	−0.6 (3)
C13—C14—C15—O1	−0.2 (3)	C38—C33—C34—C35	0.9 (3)
C19—C14—C15—C16	−0.5 (3)	C32—C33—C34—C35	178.98 (17)
C13—C14—C15—C16	−179.62 (17)	O2—C34—C35—C36	178.9 (2)
O1—C15—C16—C17	−179.35 (19)	C33—C34—C35—C36	−0.7 (3)
C14—C15—C16—C17	0.1 (3)	C34—C35—C36—C37	0.0 (3)
C15—C16—C17—C18	0.3 (3)	C33—C38—C37—C36	−0.4 (3)
C16—C17—C18—C19	−0.4 (3)	C33—C38—C37—Cl2	−179.74 (13)
C16—C17—C18—Cl1	179.50 (16)	C35—C36—C37—C38	0.6 (3)
C17—C18—C19—C14	0.0 (3)	C35—C36—C37—Cl2	179.87 (17)
Cl1—C18—C19—C14	−179.91 (14)	C14—C13—N1—C1	176.31 (15)
C15—C14—C19—C18	0.5 (3)	C6—C1—N1—C13	106.50 (19)
C13—C14—C19—C18	179.61 (16)	C2—C1—N1—C13	−76.7 (2)
C25—C20—C21—C22	−3.6 (3)	C33—C32—N2—C20	−176.22 (15)
N2—C20—C21—C22	179.64 (16)	C25—C20—N2—C32	75.3 (2)
C25—C20—C21—C29	178.15 (18)	C21—C20—N2—C32	−107.84 (19)

### Hydrogen-bond geometry (Å, º)

Cg1 and Cg3 are the centroids of the C1–C6 and C20–C25 rings, respectively.

**Table:**

*D*—H···*A*	*D*—H	H···*A*	*D*···*A*	*D*—H···*A*
O1—H1···N1	0.82	1.92	2.646 (2)	147
O2—H2···N2	0.82	1.90	2.630 (2)	147
C31—H31c···*Cg*1	0.96	2.90	3.743 (3)	147
C12—H12*A*···*Cg*3^i^	0.96	2.98	3.833 (3)	149

Symmetry code: (i) *x*−1, *y*, *z*.
